# Phylogenetic Analysis of *Trichoderma* Species Associated with Green Mold Disease on Mushrooms and Two New Pathogens on *Ganoderma sichuanense*

**DOI:** 10.3390/jof8070704

**Published:** 2022-07-03

**Authors:** Xiao-Ya An, Guo-Hui Cheng, Han-Xing Gao, Xue-Fei Li, Yang Yang, Dan Li, Yu Li

**Affiliations:** 1 Department of Plant Protection, Shenyang Agricultural University, Shenyang 110866, China; anxiaoya2016@163.com (X.-Y.A.); chengguohui2016@163.com (G.-H.C.); 2 Engineering Research Center of Chinese Ministry of Education for Edible and Medicinal Fungi, Jilin Agricultural University, Changchun 130118, China; gaohanxing168@163.com (H.-X.G.); lixuefei2020@163.com (X.-F.L.); 3 Environment and Plant Protection Institute, Chinese Academy of Tropical Agricultural Sciences, Haikou 571101, China; yyjob1992@163.com

**Keywords:** taxonomy, green mold disease, one new taxon, mycoparasites, biological agents

## Abstract

Edible and medicinal mushrooms are extensively cultivated and commercially consumed around the world. However, green mold disease (causal agent, *Trichoderma* spp.) has resulted in severe crop losses on mushroom farms worldwide in recent years and has become an obstacle to the development of the *Ganoderma* industry in China. In this study, a new species and a new fungal pathogen on *Ganoderma sichuanense* fruitbodies were identified based on the morphological characteristics and phylogenetic analysis of two genes, the translation elongation factor 1-α (TEF1) and the second-largest subunit of RNA polymerase II (RPB2) genes. The new species, *Trichoderma ganodermatigerum* sp. nov., belongs to the Harzianum clade, and the new fungal pathogen was identified as *Trichoderma koningiopsis*. Furthermore, in order to better understand the interaction between *Trichoderma* and mushrooms, as well as the potential biocontrol value of pathogenic *Trichoderma*, we summarized the *Trichoderma* species and their mushroom hosts as best as possible, and the phylogenetic relationships within mushroom pathogenic *Trichoderma* species were discussed.

## 1. Introduction

Mushrooms have been used by humans for millennia and are consumed for their nutritive and medicinal values [1,2]. Most of them are appreciated as delicacies and are extensively cultivated and commercially consumed in many countries. Some mushrooms also have high pharmacological activities, especially *Ganoderma* spp. [3,4]. *Ganoderma sichuanense*, described from China and previously confused with *G. lucidum*, an oriental fungus, has a long history in China, Japan, and other Asian countries for promoting health and longevity [5,6]. The mushroom is famous for its pharmacological effects [7,8] and is widely cultivated in northeastern China. However, *Trichoderma* green mold diseases have increased and pose a serious threat to its production [9,10,11].

*Trichoderma* has been studied for more than 200 years since it was established by Persoon in 1794 [12], while sharp development occurred in the past few decades, when a large number of taxonomic articles were published [13,14,15,16,17,18,19,20,21,22,23,24,25,26]. At present, similar to *Fusarium*, *Aspergillus*, or *Penicillium*, *Trichoderma* is a species-rich genus [15] and has been segregated into many groups or clades based on the phylogenetic relationships within the genus [27,28,29]. Moreover, the rapid development of *Trichoderma* is inseparable from its various uses. For example, it can not only be used as a highly efficient producer of plant biomass-degrading enzymes for biofuel and other industries, but also as a very effective biological agent for plant disease management [30,31,32,33]. Furthermore, *Trichoderma* has also been an initially produce white and dense mycelia highly similar to mushroom mycelia, which makes it difficult to distinguish them, causing the best period of control to be missed. Thus, it is particularly important to explore the specificity of *Trichoderma* species and the interaction between *Trichoderma* and its host for disease control.

Between 2020 and 2021, during fieldwork at mushroom cultivation bases, we found that green mold disease occurred continuously in *G. sichuanense* production areas in the following provinces of China: Heilongjiang, Jilin, and Shandong, leading to a significant negative effect on the development of fruitbodies. We collected diseased specimens and isolated the pathogens from several bases and identified them based on molecular and morphological characteristics. A new *Trichoderma* species and a new host record were confirmed. In addition, we summarized the *Trichoderma* species reported on mushrooms as best as possible and provided their recorded hosts. The relationships among these species were also discussed by constructing a phylogeny tree with multi-locus data, which is expected to help us know more about the relationships between *Trichoderma* species and their hosts, and to help search for *Trichoderma* species with potential biocontrol value.

## 2. Materials and Methods

### 2.1. Fungal Isolation

Diseased samples of *G. sichuanense* were collected from Jilin, Heilongjiang, and Shandong Provinces, China, and deposited in the Herbarium of Mycology, Jilin Agricultural University (HMJAU). Diseased tissues were cut into small pieces (5 mm × 5 mm × 5 mm) using a sterilized scalpel, immersed in 75 percent alcohol for 45 s before being rinsed three times with sterilized water, and placed onto Potato Dextrose Agar (PDA, BD, USA) plates containing 100 mg/L of streptomycin sulfate (Solarbio, Bejing, China), and then incubated at room temperature. Pure cultures were obtained using single-spore isolates following the method described by Chomnuti et al. [34]. Germinated spores were transferred to fresh PDA plates and incubated at 25 °C for one or two weeks. Living cultures were deposited in the Engineering Research Center of Edible and Medicinal Fungi, Ministry of Education, Jilin Agricultural University (Changchun, Jilin, China).

### 2.2. Growth Characterization

Colony characteristics, growth rates, and optimum temperatures for growth were determined according to the methods of Jaklitsch [18,19] by using agar media cornmeal dextrose agar (CMD, 40 g cornmeal + 2% (*w*/*v*) dextrose (Genview, Beijing, China) + 2% (*w*/*v*) agar (Genview, Beijing, China)), PDA, and synthetic low nutrient agar (SNA, pH adjusted to 5.5) [35]. Colonies were incubated in 9 cm diameter Petri dishes at 25 °C with alternating 12 h/12 h fluorescent light/darkness and measured daily until the dishes were covered with mycelia. The influence of temperature on growth was studied by growing isolates on PDA, SNA, and CMD at 15 °C, 20 °C, 25 °C, 30 °C, and 35 °C under dark conditions. Each temperature was repeated for five plates, and the experiment was repeated three times. 

### 2.3. Morphological Study

The characteristics of asexual states were described following the methods of Jaklitsch [36] and Rifai [37]. Microscopic observations were conducted using a Zeiss Axio Lab A1 light microscope (Göttingen, Germany) (objectives 10, 20, 40, and 100 oil immersion). All measurements and photographs were performed using a Zeiss Imager A2 microscope with an Axiocam 506 color camera and integrated software. Microscopically, the characteristics of 50 conidia and conidiophores from the isolates were observed. The effects of *Trichoderma* on *Ganoderma* morphology were studied using a Hitachi, model SU8010, Field Emission Scanning Electron Microscope (FESEM) at Jilin Agricultural University.

### 2.4. DNA Extraction, PCR, and Sequencing

Mycelia were harvested from three-day-old cultures on PDA for DNA extraction according to the manufacturer’s instructions (NuClean Plant Gen DNA Kit, CWBIO, Taizhou, China). Sequences of ITS, TEF1, and RPB2 genes were amplified by polymerase chain reaction (PCR) with the pairs of primers ITS4 (5′-TCCTCCGCTTATTGATATGC-3′) and ITS5 (5′-GGAAGTAAAAGTCGTAACAAGG-3′) [38], primers EF1-728F (5′-CATCGAGAAGTTCGAGAAGG-3′) [39] and TEF1-LLErev (5′-GCCATCCTTGGAGATACCAGC-3′) [40], and primers RPB2-5F (5′-GAYGAYMGWGATCAYTTYGG-3′) and RPB2-7CR (5′-CCCATRGCTTGYTTRCCCA-3′) [41], respectively.

PCR was carried out in a 25 μL reaction mixture containing 1 μL of DNA sample, 12.5 μL 2 × SanTaq PCR Mix (Sangon Biotech, Shanghai, China), 1 μL of each primer (10 µM), and 9.5 μL nuclease-free water. The PCR conditions were as follows: initial denaturation at 94 °C for 3 min, then denaturation at 94 °C for 30 s, annealing for 45 s with the corresponding temperatures (56 °C for TEF1, and 55 °C for RPB2), extension at 72 °C for 1 min, followed by 35 cycles, then a final extension at 72 °C for 10 min, using an Applied Biosystems S1000 ^TM^ Thermal Cycler machine. PCR products were sent to the Changchun Branch of Sangon Biotech Co., Ltd. (Changchun, China) for paired-end sequencing, and the results were first assembled using BioEdit [42] and then confirmed by BLAST on NCBI (https://blast.ncbi.nlm.nih.gov/Blast.cgi, accessed on 21 June 2021).

### 2.5. Phylogenetic Analyses

BLASTn searches with the sequences were performed against NCBI to detect the most closely related species (http://www.blast.ncbi.nlm.nih.gov/, accessed on 22 December 2021). Phylogenetic trees were constructed using TEF1 and RPB2 sequences, and phylogenetic analyses were performed with the Maximum Likelihood (ML), Maximum Parsimony (MP), and Bayesian Inference (BI) methods. New sequences were generated from the new species in this study, along with reference sequences retrieved from GenBank (Table 1). The *Trichoderma* sequences associated with mushroom green mold are listed in Table 2. Multiple alignments of all common sequences and reference sequences were automatically generated using MAFFT V.7.471 [43], with manual improvements made using BioEdit when necessary [42], and converted to nexus and NEX format through the software Aliview [44]. In the analysis, ambiguous areas were excluded and gaps were regarded as missing data.

An MP phylogram was constructed with PAUP 4.0b10 [106] from the combined sequences of TEF1 and RPB2, using 1000 replicates of a heuristic search with random addition of sequences and subsequent tree bisection and reconnection (tbr) branch swapping. Analyses were performed with all characters treated as unordered and unweighted, and gaps treated as missing data. The topological confidence of the resulting trees was tested by maximum parsimony bootstrap proportion (MPBP) with 1000 replications, each with 10 replicates of random addition of taxa. An ML phylogram was constructed with Raxmlgui 2.0 [107] with the sequence after alignment. The ML + Rapid bootstrap program and 1000 repeats of the GTRGAMMAI model were used to evaluate the bootstrap proportion (BP) of each branch for constructing the phylogenetic tree. The BI analysis was conducted using MrBayes 3.2.7 [108] using a Markov Chain Monte Carlo (MCMC) algorithm. Nucleotide substitution models were determined using MrModeltest 2.3 [109]. The best model for combined sequences was HKY + I + G.

## 3. Results

### 3.1. Molecular Phylogeny

**Figure 1:**
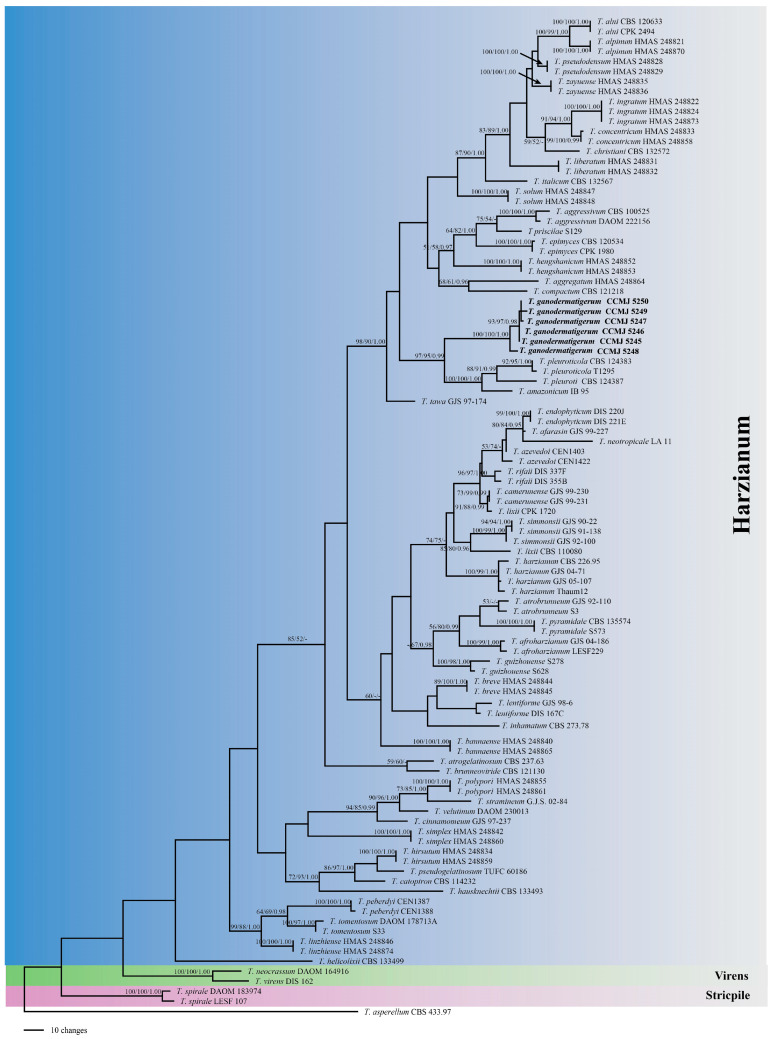
Phylogeny of *Trichoderma* using MP analysis based on combined TEF1 and RPB2 sequences. MPBP ≥ 50%, MLBP ≥ 50%, and BIPP ≥ 0.9 are shown on the branches (MPBP/MLBP/BIPP). The sequences in bold are the new species.

**Figure 2:**
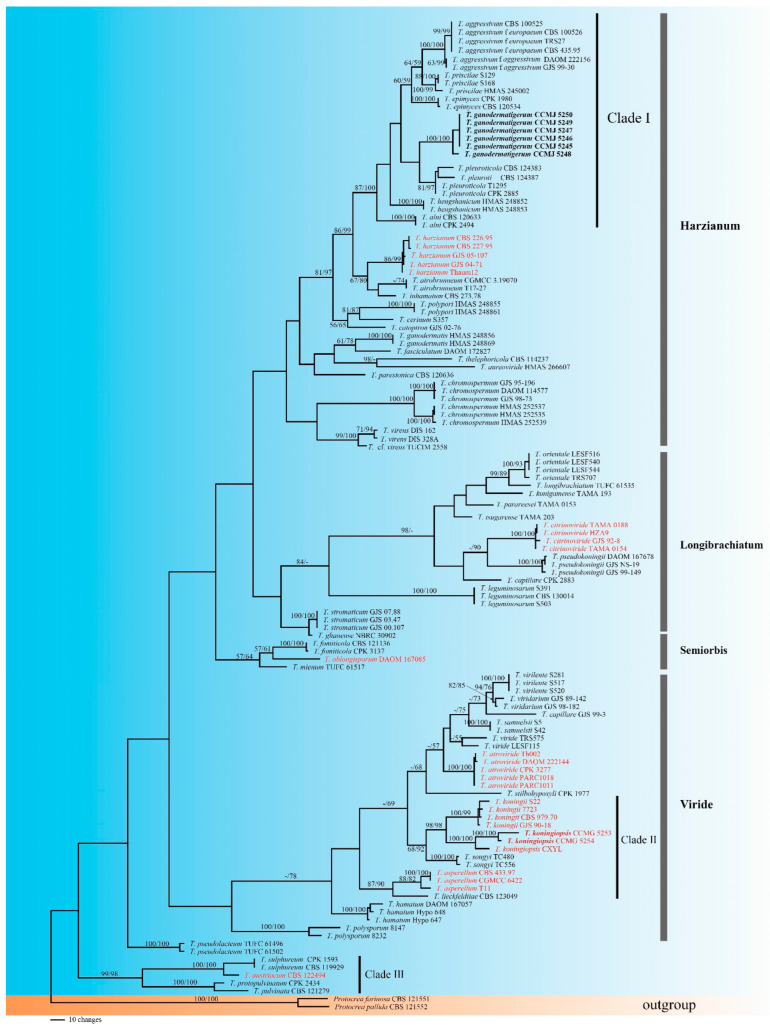
Phylogeny of *Trichoderma* associated with mushrooms using MP analysis based on concatenated TEF1 and RPB2 sequences. Branches are labeled with MPBP ≥ 50% and MLBP ≥ 50%. The biological agents are marked in red, and the new sequences in this study are in bold.

Species recognition: The dataset for the new species phylogenetic analyses included sequences from 100 taxa (Table 1). Multi-locus data were concatenated, which comprised 2321 characters, with TEF1 1293 characters and RPB2 1028 characters. Estimated base frequencies were as follows: A = 0.231650, C = 0.281772, G = 0.234671, and T = 0.251907; substitution rates were as follows: AC = 1.069464, AG = 4.197119, AT = 0.935747, CG = 0.993621, CT = 4.979475, and GT = 1.000000. The MP and ML trees showed similar topologies with high statistical support values. The MP tree was selected as the representative phylogeny. In Bayesian analysis, the average standard deviation of split frequencies at the end of the total MCMC generations was calculated as 0.008946, which is less than 0.01. Most of the tree topologies resulting from three analyses were nearly the same. In the resulting tree (Figure 1), the combined phylogenetic analyses using TEF1-α and RPB2 showed that the six strains of *T. ganodermatigerum* represent phylogenetically distinct species with high statistical supports (MPBP/MLBP/BIBP = 100%/100%/1.0), and clustered together with the species in the Harzianum clade [16]. The new species is most related to the clade that contains *T. amazonicum*, *T. pleuroticola*, *T. hengshanicum*, and *T.* *pleuroti*. Two collections of CCMJ5253 and CCMJ5254 clustered with *T. koningiopsis* with high support (MPBP/MLBP = 100/100) (Figure 2).

Phylogenetic structure: Some sections could be found among the *Trichoderma* strains associated with mushrooms and are mainly concentrated in the Harzianum clade (Figure 2). *Trichoderma longibrachiatum*, *T. citrinoviride*, *T. pseudokoningii*, and *T. ghanense* are from section *Longibrachiatum*, whose members are best known as producers of cellulose-hydrolyzing enzymes [74,110,111]. *Trichoderma atroviride*, *T. viride*, *T. koningii*, *T. hamatum*, *T. minutisporum*, *T. polysporum*, *T. viride*, and *T. asperellum* are from section *Trichoderma* or the Viride clade [36,111].

The phylogenetic structure according to ecology: Species in the Harzianum clade are commonly fungicolous, living in different types of habitats [112,113]. They are most commonly isolated from soil or found on decomposing plant material where they occur cryptically or parasitize other fungi [18,53,114], and those species are possibly the most common endophytic “species” in wild trees [115,116]. There is usually no apparent host specialization [117]. However, some exceptions to this trend exist. Clade I in the Harzianum clade of the tree is a collection of species with relatively narrow host ranges, or in other words, a strong host preference. *Trichoderma atrobrunneum* was found in soil or on decaying wood, clearly or cryptically parasitizing other fungi. *Trichoderma pleuroti*, just like *T. aggressivum*, has thus far never been isolated from areas outside of mushroom farms [118]. Furthermore, *T. epimyces* has only been reported on *Polyporus umbellatus* [49], and *T. priscilae* has been reported from basidiomes of *Crepidotus* and *Stereum* [20].

Some other species such as *T. atroviride*, *T. asperellum*, *T. harzianum*, and *T. longibrachiatum* were also found in significant proportions in *Agaricus* compost [119]. *Trichoderma stromaticum* and its *Hypocrea* teleomorph are only known from cocoa and are often associated with tissue infected with the basidiomycetous pathogen *Crinipellis perniciosa* [55].

Although some of these pathogenic *Trichoderma* species (e.g., species gathered in or near Clade II) have been explored as biocontrol agents for plant diseases, *T. atroviride*, *T. viride*, *T. koningii*, *T. koningiopsis*, and *T. asperellum* serve as pathogens with broad host ranges on mushrooms. *Trichoderma sulphureum*, *T. protopulvinatum*, *T. pulvinatum*, and *T. austriacum* coalesce into a subclade (Clade III), and each of these species has been reported on a particular fungus [18,19].

### 3.2. Taxonomy

**Figure 3:**
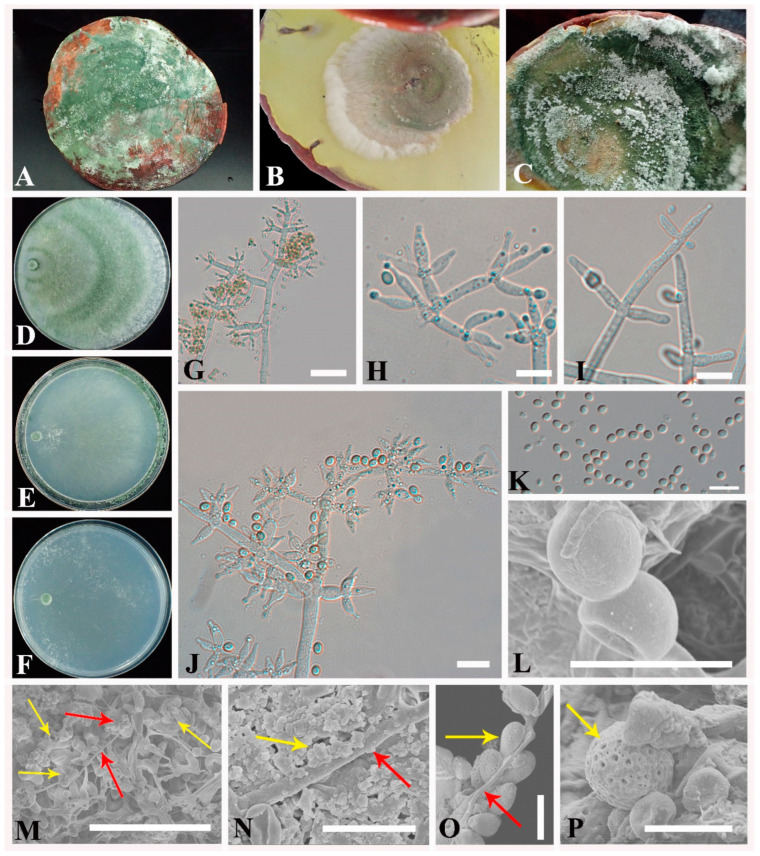
Morphological characteristics of *T. ganodermatigerum*. (**A**–**C**) diseased fruitbody; (**D**–**F**) colony on PDA, CMD, and SNA; (**G**–**J**) conidiophores and phialides; (**K**,**L**) conidia; (**M**–**P**) interactions of *G. sichuanense* and *T. ganodermatigerum*; (**M**) *Trichoderma* hyphae and conidia are filled in the *Ganoderma* tissue, causing the tissue to become rough or even depressed; (**N**) *Trichoderma* hyphae covered with *Ganoderma* tissue; (**O**) clinged *Trichoderma* hyphae and healthy *Ganoderma* spores; (**P**) abnormal *Ganoderma* spores in diseased tissue. Bars: G, Q = 20 µm; H–J, M–P = 10 µm; K = 50 µm; L = 5 µm. The yellow arrows indicate the tissues and spores of *G. sichuanense*, and the red arrows indicate the hyphae and spores of *T. ganodermatigerum*.

*Trichoderma ganodermatigerum* X.Y. An & Y. Li, sp. nov. Figure 3A–L.

MycoBank: MB 843898.

Diagnosis: Phylogenetically, *T. ganodermatigerum* formed a distinct clade and is related to *T. amazonicum* (Figure 1). Both *T. amazonicum* and *T. ganodermatigerum* form dense concentric rings, pyramidal branching patterns, and branches toward the tip; mycelium grows slowly or does not grow at 35 °C; conidia globose, smooth, and green. As for *T. amazonicum*, there is no diffusing pigmentation on CMD media and a slightly fruity odor; a brown diffusing pigmentation of the agar is formed in some strains on PDA media [50]. Phylogenetic analysis of TEF1 and RPB2 gene sequences also revealed that *T. ganodermatigerum* was phylogenetically distinct not only from *T. amazonicum* but also from other previously reported *Trichoderma* species.

Etymology: The name refers to the host genus “*Ganoderma*” from which it was isolated.

Typification: CHINA. Jilin Province, Panshi City, Songshan County, from *Ganoderma sichuanense*, alt. 310 m, 126°56′ E, 42°77′ N, 18 August 2021, *Xiaoya An*, HMJAU59014, preserved in Engineering Research Center of Chinese Ministry of Education for Edible and Medicinal Fungi of Jilin Agricultural University. Ex-type culture CCMJ5245. Sexual morph: Undetermined. (ITS: ON399102, TEF1: ON567195, and RPB2: ON567189).

Teleomorph: Unknown.

Description: The optimum temperature was 25 °C, and the colony radius on CMD was 7–9 mm at 15 °C, 19–23 mm at 20 °C, 43–52 mm 25 °C, and 32–36 mm at 30 °C, with no growth at 35 °C, and mycelium covering the plate after ten days at 25 °C (Figure 3E). Colony hyaline, thin, and radiating, white in the initial stage, and gradually turned to light green with slight zonate. Mycelia were sparse and delicate, hard to be observed, and aerial hyphae were inconspicuous. Conidiation starting after six days, formed in pustules. Pustules were spreading near the original inoculum or at the edge of the colony, distributed loosely in the plate, white in the initial stage and then turned green. No chlamydospores were observed. No distinct odor and no diffusing pigment were observed.

Colony radius on SNA after 72 h 5–8 mm at 15 °C,13–15 mm at 20 °C, 42–43 mm at 25 °C, and 25–28 mm at 30 °C, and can hardly see the growth at 35 °C. Mycelium covering the plate after six days at 25 °C (Figure 3F). Colony hyaline, thin, irregular, surface mycelium scant. Aerial hyphae are inconspicuous and short. Conidiation starting after three days, formed in loose pustules. Pustules initially white, loose distribution, later turn aggregated and green. No chlamydospores were observed. No distinct odor and no diffusing pigment were observed.

On PDA, the colony radius was 9–12 mm at 15 °C, 22–28 mm at 20 °C, 38–44 mm at 25 °C, and 30–40 mm at 30 °C, with no growth at 35 °C after 72 h, and mycelium covering the plate after 5–6 days at 25 °C (Figure 3D). The colony was circular, spreading in several concentric rings; aerial hyphae were common, dense, and green; the margin was relatively loose and whitish under the alternative light situations. However, mycelia were aerated and white, and only green appeared near the inoculation site under the condition of total darkness. Conidiation starting after 3–4 days, formed on aerial hyphae, spreading in a circle around the original inoculum. Conidiophores are typically tree-like, straight, or slightly curved, comprising a distinct main axis with side branches paired or unilateral and often terminating in whorls of 3–4 divergent phialides, rarely with a terminal solitary phialide (Figure 3G–J), branches densely disposed, arising at mostly vertical angles upwards, rebranching 1–3 times; the distance between two neighboring branches is (6.6–) 10.0–30.0 (–35.6) μm. Phialides formed paired or in whorls of 3–5, lageniform, spindly, usually arising at an acute angle to the axis, rarely solitary (Figure 3F), (1.1–) 2.8–12.3 (–16) μm× (0.2–) 1.9–3.4 (–3.6) μm, *l/w* ratio (1.6–) 1.7–5.9 (–7.0), (0.2–) 1.4–2.6 (–2.8) μm wide at the base. Conidia one-celled, green, smooth-walled, globose to subglobose, sometimes ellipsoid, (3.4–) 3.6–4.8 (–5.3) μm× (2.9–) 3.2–4.3 (–4.6) μm, *l/w* ratio 1.1–1.5. No chlamydospores were observed. No distinct odor and no diffusing pigment were observed.

Distribution: Jilin, Shandong, and Heilongjiang Provinces, China.

Additional specimen examined: China, Jilin Province, Panshi city, Songshan County, from *Ganoderma sichuanense*, alt. 310 m, 126°56′ E, 42°77′ N, 11 Oct. 2021, *Xiaoya An*, HMJAU59013.

Notes: Fungicolous on the fruiting body of *G. sichuanense* in terrestrial habitats. It produces extremely tree-like main axes and branches and green, globose conidia (Figure 3N). The results of the phylogenetic tree strongly support its status as a new taxon (Figure 1), indicating its affinity to the Harzianum clade [16]. The species was related to *T. amazonicum* and *T. pleuroticola*. Regarding *T. amazonicum*, it is a host-specific endophyte and might have potential for biocontrol of *Hevea* diseases [50]. Phylogenetically, *T. ganodermatigerum* is related to *T. pleuroticola* in the mycoparasite group. Morphologically, both species grow rapidly and form broad concentric rings on PDA. Conidiation formed small pustules, and the green spores cause the colony to change from light to dark green [120]. The difference is that the new species starts with white, aerial mycelia and spores are more spherical or nearly spherical, with obvious green color, while the spores of *T. pleuroticola* are light green, subglobose to broadly ellipsoidal conidia, slightly smaller than *T. ganodermatigerum*, and reported more on *Pleurotus ostreatus*, *Pleurotus eryngii* var. *ferulae*, *Lentinula edodes*, and *Cyclocybe aegerita* [69,73,83,120].

*Trichoderma koningiopsis* Samuels, Carm. Suárez & H.C. Evans 2006.

Description: Fungicolous, colonized the fruiting body of *G. sichuanense*, causing green mold disease and occurring mostly from June to September. It is very difficult to distinguish the mycelium in the early stage, and only scattered spots present under the cap. Then, white mycelium appeared, with radiating growth. The edge of the colony is often accompanied by a yellow or brown line. A large number of green spores were produced in the late stage. Young basidiomes were inoculated with *T. koningiopsis*, which reproduced the original signs; the same pathogen was isolated again from the diseased fruitbody.

On PDA, the colony was radial, first whitish, became dark green with fluffy hyphae after ten days. Aerial hyphae were common and dense, but no concentric rings were observed. Mycelia often appear white in complete darkness, and light stimulates spore production, resulting in a green colony. Conidia formed in pustules, spreading near the original inoculum, white, turning green later. On CMD, mycelium covering the plate after ten days at 25 °C, loose and slim, aerial hyphae were absent. Conidia were formed in pustules, which were only produced at the edge of a colony. On SNA media, concentric rings of light yellow or green appeared, and spores were produced in four days. Conidiophore branches arose at right angles, and primary branches arose singly or in pairs. Conidia were ellipsoidal to oblong-shaped, green, 2.8–7.3 × 2.5–7.0 µm. No chlamydospores, no distinct odor, and no diffusing pigment were observed.

Material examined: CHINA, Jilin Province, on a fruiting body of *Ganoderma*, 4 August 2020; *Xiaoya An*, HMJAU59012, living culture CCMJ5253, CCMJ5254 (ITS: ON385996, ON385947; TEF1: ON567187, ON567188, and RPB2: ON567201, ON567202, respectively).

Notes: *Trichoderma koningiopsis* is found throughout tropical America, as well as East Africa, Europe, Canada, and eastern North America [23]. This species is mainly found in soil, twigs, and decayed leaves, and the sexual type is mostly found in wood. At present, *T. koningiopsis* has been reported to cause green mold of *Phaiius rubrovolvata* [91], and to our knowledge, this is the first time that it has caused green mold on *G. sichuanense*. Our sequences had high similarity to the *T. koningiopsis* sequence after BLAST, and the results of the phylogenetic tree also confirmed the correctness of the classification (Figure 2).

## 4. Discussion

Edible and medicinal mushrooms have become a very important crop and are grown commercially in many countries [1,121], but the production, including the yield and quantity, is challenged by fungal diseases [2,24]. *Trichoderma ganodermatigerum* is a new species of *Trichoderma*. The results from the phylogenetic analyses separate the new species from other closely related and morphologically similar species. The sequences indicate it belongs to the Harzianum clade. To date, more than forty *Trichoderma* species have been reported to be associated with mushroom green mold disease. *Trichoderma atroviride*, *T. harzianum*, *T. koningii*, *T. longibrachiatum*, *T. pseudokoningii*, and *T. viride* are the six most commonly cited species causing disease on edible mushrooms (Table 2), all of which could infect six to eleven species of cultivated mushrooms [61,64,68,73,83,91,119,122,123]. Before this study, there were seven known species that could cause *G. sichuanense* diseases, namely, *T. koningii, T. longibrachiatum*, *T. pseudokoningii*, *T. viride*, *T. atrobrunneum*, *T. ganodermatis* [47], and *T. hengshanicum* [87], while *T. orientale* can cause disease on *G. applanatum* [124].

*Trichoderma* green mold infection in edible basidiomycetes has a long history [125]. There are many types of interactions between mushrooms and *Trichoderma* [126,127,128,129]. Similar to *T. aggressivum*, the causal agent of *Agaricus* green mold disease [130], no obvious biting phenomenon was observed between pathogen and mushroom in this study. Through SEM observation, in the interaction zone between *G. sichuanense* and *T. ganodermatigerum*, the tissue surface of *Ganoderma* became uneven with irregular holes (Figure 3K), the pores on the *Ganoderma* spores became larger, and the double-layer structure was damaged, resulting in spore invagination (Figure 3L), which was similar to the interaction between *Trichoderma* and shiitake [83]. We can at least suspect that the cell-wall-degrading enzymes play an important role in the process according to the symptoms of soft tissue with holes or even oozing liquid of *Ganoderma*. In addition, *T. songyi* could have great biological potential because it is closely related to the biological agents (Figure 2, Clade II).

The application of the *Trichoderma* species as biocontrol agents began in 1934 when Weindling first discovered that *Trichoderma* could be parasitic on the hyphae of *Rhizoctonia solani*, and since then, an increasing amount of research has focused on this field [131]. Because many *Trichoderma* species are symbiotic and fungal parasitoids, they need to produce degradation enzymes or secondary metabolites to obtain nutrients from the host, so they have been developed as biocontrol agents for plant diseases [50,55,112,132,133]. Among the species associated with mushrooms, nine species are used as biological agents already. *Trichoderma koningiopsis*, the new pathogen for *G. sichuanense* in this study, has been a biocontrol agent for a long time [134]. Since *T. ganodermatigerum* can infect cultivated *Ganoderma*, leading to growth stagnation or the cessation of sporulation of *Ganoderma*, it could be a potential biocontrol agent for plant disease. Therefore, the parasitic characteristics and compounds should be further studied.

## Figures and Tables

**Table 1:** Strain information and GenBank accession numbers of sequences used for phylogenetic analyses for new species.

Species	Strains	GenBank Accession Number		References
		TEF1	RPB2	
*T. afarasin*	GJS 99-227	AF348093	—	[45]
*T. afroharzianum*	LESF229	KT279013	KT278945	[46]
*T. afroharzianum*	GJS04-186 (T)	FJ463301	FJ442691	In GenBank
*T. aggregatum*	HMAS248864	KY688063	KY688002	[47]
*T. aggressivum*	CBS100525	AF534614	AF545541	[48]
*T. aggressivum*	DAOM222156	AF348098	FJ442752	[45]
*T. alni*	CPK2494	EU498313	EU498350	[49]
*T. alni*	CBS120633 = CPK1982 (T)	EU498312	EU498349	[49]
*T. alpinum*	HMAS248870	KY688017	KY687963	[47]
*T. alpinum*	HMAS248821 (T)	KY688012	KY687958	[47]
*T. amazonicum*	IB95	HM142377	HM142368	[50]
*T. asperellum*	CBS433.97 = TR3 (T)	AF456907	EU248617	[51]
*T. atrobrunneum*	S3	KJ665376	KJ665241	[20]
*T. atrobrunneum*	GJS92-110 (T)	AF443942	—	[16]
*T. atrogelatinosum*	CBS237.63 (T)	—	KJ842201	In GenBank
*T. azevedoi*	CEN1403	MK696638	MK696800	[52]
*T. azevedoi*	CEN1422	MK696660	MK696821	[52]
*T. bannaense*	HMAS248865	KY688038	KY688003	[47]
*T. bannaense*	HMAS248840 (T)	KY688037	KY687979	[47]
*T. breve*	HMAS248845	KY688046	KY687984	[47]
*T. breve*	HMAS248844 (T)	KY688045	KY687983	[47]
*T. brunneoviride*	CBS121130 = CPK2014	EU498316	EU498357	[49]
*T. camerunense*	GJS99-231	AF348108	—	[45]
*T. camerunense*	GJS99-230 (T)	AF348107	—	[45]
*T. catoptron*	GJS02-76 = CBS114232 (T)	AY391963	AY391900	[53]
*T. christiani*	CBS132572 = S442 (T)	KJ665439	KJ665244	[20]
*T. cinnamomeum*	GJS97-237 (T)	AY391979	AY391920	[53]
*T. compactum*	CBS121218	KF134798	KF134789	[54]
*T. concentricum*	HMAS248858	KY688028	KY687997	[47]
*T. concentricum*	HMAS248833 (T)	KY688027	KY687971	[47]
*T. endophyticum*	DIS220J	FJ463330	FJ442690	[55]
*T. endophyticum*	DIS221E	FJ463316	FJ442775	In GenBank
*T. epimyces*	CPK1980	EU498319	EU498359	[49]
*T. epimyces*	CBS120534 = CPK1981 (T)	EU498320	EU498360	[49]
***T. ganodermatigerum***	**CCMJ5245 (T)**	**ON567195**	**ON567189**	**This study**
***T. ganodermatigerum***	**CCMJ5246**	**ON567196**	**ON567190**	**This study**
***T. ganodermatigerum***	**CCMJ5247**	**ON567197**	**ON567191**	**This study**
***T. ganodermatigerum***	**CCMJ5248**	**ON567198**	**ON567192**	**This study**
***T. ganodermatigerum***	**CCMJ5249**	**ON567199**	**ON567193**	**This study**
***T. ganodermatigerum***	**CCMJ5250**	**ON567200**	**ON567194**	**This study**
*T. guizhouense*	S278	KF134799	KF134791	[54]
*T. guizhouense*	S628	KJ665511	KJ665273	[20]
*T. harzianum*	GJS05-107	FJ463329	FJ442708	In GenBank
*T. harzianum*	GJS04-71	FJ463396	FJ442779	In GenBank
*T. harzianum*	Thaum12	MT081433	MT118248	In GenBank
*T. harzianum*	CBS226.95 (T)	AF534621	AF545549	[48]
*T. hausknechtii*	Hypo649 = CBS133493 (T)	KJ665515	KJ665276	[20]
*T. helicolixii*	S640 = CBS133499 (T)	KJ665517	KJ665278	[20]
*T. hengshanicum*	HMAS248853	KY688055	KY687992	[47]
*T. hengshanicum*	HMAS248852 (T)	KY688054	KY687991	[47]
*T. hirsutum*	HMAS248859	KY688030	KY687998	[47]
*T. hirsutum*	HMAS248834 (T)	KY688029	KY687972	[47]
*T. ingratum*	HMAS248824	KY688019	KY687964	[47]
*T. ingratum*	HMAS248873	KY688022	KY688010	[47]
*T. ingratum*	HMAS248822 (T)	KY688018	KY687973	[47]
*T. inhamatum*	CBS273.78 (T)	AF348099	FJ442725	[45]
*T. italicum*	S131 = CBS132567 (T)	KJ665525	KJ665282	[20]
*T. lentiforme*	DIS167C	FJ463309	FJ442689	In GenBank
*T. lentiforme*	GJS98-6 (T)	AF469195	—	[16]
*T. liberatum*	HMAS248832	KY688026	KY687970	[47]
*T. liberatum*	HMAS248831 (T)	KY688025	KY687969	[47]
*T. linzhiense*	HMAS248874	KY688048	KY688011	[47]
*T. linzhiense*	HMAS248846 (T)	KY688047	KY687985	[47]
*T. lixii*	CBS110080 = GJS97-96	FJ716622	KJ665290	[20]
*T. neocrassum*	DAOM164916 = CBS336.93 (T)	AF534615	AF545542	[48]
*T. neotropicale*	LA11	HQ022771	—	[56]
*T. peberdyi*	CEN1387	MK696619	MK696781	[52]
*T. peberdyi*	CEN1388	MK696620	MK696782	[52]
*T. pleuroticola*	T1295	EU279973	—	[57]
*T. pleuroticola*	CBS124383 (T)	HM142381	HM142371	[50]
*T. pleuroti*	CBS124387 (T)	HM142382	HM142372	[50]
*T. polypori*	HMAS248855	KY688058	KY687994	[47]
*T. polypori*	HMAS248861	KY688059	KY688000	[47]
*T. priscilae*	S129	KJ665689	KJ665332	[20]
*T. pseudodensum*	HMAS248829	KY688024	KY687968	[47]
*T. pseudodensum*	HMAS248828 (T)	KY688023	KY687967	[47]
*T. pseudogelatinosum*	TUFC60186 (T)	JQ797397	JQ797405	[58]
*T. pyramidale*	S573	KJ665698	—	[20]
*T. pyramidale*	S73 = CBS135574 (T)	KJ665699	KJ665334	[20]
*T. rifaii*	DIS337F	FJ463321	FJ442720	In GenBank
*T. rifaii*	DIS355B (T)	FJ463324	—	In GenBank
*T. simmonsii*	GJS90-22	AY391984	AY391925	[53]
*T. simmonsii*	GJS92-100	AF443937	FJ442710	[16]
*T. simmonsii*	GJS91-138	AF443935	FJ442757	[16]
*T. simplex*	HMAS248860	KY688042	KY687999	[47]
*T. simplex*	HMAS248842 (T)	KY688041	KY687981	[47]
*T. solum*	HMAS248848	KY688050	KY687987	[47]
*T. solum*	HMAS248847 (T)	KY688049	KY687986	[47]
*T. spirale*	DAOM183974	EU280049	—	[57]
*T. spirale*	LESF107	KT279022	KT278956	[46]
*T. stramineum*	GJS02-84 = CBS114248 (T)	AY391999	AY391945	[53]
*T. tawa*	GJS97-174 = CBS114233 (T)	AY392004	AY391956	[53]
*T. tomentosum*	S33	KF134801	KF134793	[54]
*T. tomentosum*	DAOM178713A (T)	AF534630	AF545557	[48]
*T. velutinum*	DAOM230013 = CPK298	AY937415	KF134794	[59]
*T. virens*	DIS162	FJ463367	FJ442696	In GenBank
*T. zayuense*	HMAS248836	KY688032	KY687975	[47]
*T. zayuense*	HMAS248835 (T)	KY688031	KY687974	[47]

New sequences are shown in bold. The type sequences are marked with (T).

**Table 2:** Isolates and GenBank accession numbers of *Trichoderma* species associated with green mold on mushrooms.

Species	Host Range	Isolates	GenBank Accession Number		References
			TEF1	RPB2	
*T. aggressivum*	*Agaricus bisporus*	CBS100525	AF534614	AF545541	[48]
*T. aggressivum* *f. aggressivum*	*Agaricus bisporus*	GJS99-30	AF348109	—	[60]
		DAOM222156	AF348098	FJ442752	[45]
*T. aggressivum* *f. europaeum*	*Agaricus bisporus*	CBS100526 (T)	KP008993	KP009166	[45]
	—	TRS27	KP008994	KP009163	In GenBank
	—	CBS435.95	KP008998	KP009169	In GenBank
*T. alni*	*Macrotyphula* cf. *contorta*	CBS120633	EU498312	EU498349	[49]
		CPK2494	EU498313	EU498350	
*T. asperellum*	*Pleurotus ostreatus*	T11 (ACCC32725)	MF049065	—	[61]
	*Pleurotus eryngii*	—	—	—	[62]
	—	CGMCC6422	KF425756	KF425755	[63]
	—	CBS433.97 = TR3 (T)	AF456907	EU248617	In GenBank
*T. atrobrunneum*	*Ganoderma sichuanense*	CGMCC3.19070	MH464779	—	[64]
	—	T17-27	MW232537	MW232508	[65]
*T. atroviride*	*Pleurotus ostreatus*	CPK3277	EU918154	—	[66]
	*Ganoderma sichuanense*	2015005	—	—	[10]
	*Agaricus bisporus*	T33	—	—	[67]
	*Lentinula edodes*	T25	—	—	[68]
	*Pleurotus eryngii*	—	—	—	[69]
	—	PARC1011	MT454114	MT454130	[70]
	—	PARC1018	MT454121	MT454137	
	—	DAOM222144	AF456889	FJ442754	[71]
	—	Th002	AB558906	AB558915	[72]
*T. aureoviride*	*Pleurotus ostreatus*	HMAS266607	KF923280	KF923306	[73]
*T. austriacum*	*Peziza* sp.	CBS122494 (T)	FJ860619	FJ860525	[19]
*T. capillare*	*Agaricus bisporus*	CPK2883	JN182283	JN182312	[74]
		GJS99-3	JN175584	JN175529	
*T. catoptron*	*Aphyllophorales* s. l.	GJS02-76 (T)	AY391963	AY391900	[53]
*T. cerinum*	*Lentinula edodes*	S357	KF134797	KF134788	[75]
*T. chromospermum*	black mycelium and black pyrenomycete	GJS95-196	AY391975	AY391914	[53]
		GJS98-73	AY391976	AY391915	
		GJS94-68 = CBS114577	—	AY391913	
	—	HMAS252537	KF729986	KF730004	[25]
	—	HMAS252539	KF923287	KF923314	
	—	HMAS252535	KF923292	KF923315	
*T. citrinoviride*	*Lentinula edodes*	TAMA0154	AB807641	AB807653	[76]
	*Pleurotus ostreatus*	GJS92-8	JN175595	JN175544	[77]
	*Pleurotus eryngii*	GJS01-364	AY225860	AF545565	[69]
	Polypore mushroom	TAMA0188	AB807644	AB807656	[76]
	—	HZA9	MK850831	MK962804	[78]
*T. epimyces*	*Polyporus umbellatus*	CPK1980	EU498319	EU498359	[49]
		CBS120534 (T)	EU498320	EU498360	
*T. erinaceum*	—	DIS7	DQ109547	EU248604	[79]
*T. fasciculatum*	*Hypocrea* ascospores	CBS118.72	—	—	[80]
	—	DAOM172827	AF534628	AF545555	[48]
*T. fomiticola*	*Fomes fomentarius*	CBS121136	FJ860639	FJ860538	[18]
		CPK3137	FJ860640	FJ860539	
*T. ghanense*	*Agaricus bisporus*	NBRC30902	AB807638	AB807650	[76]
*T. ganodermatis*	*Ganoderma sichuanense*	HMAS248856	KY688060	KY687995	[47]
		HMAS248869	KY688061	KY688007	[47]
***T. ganodermatigerum***	***Ganoderma sichuanense***	**CCMJ5245(T)**	**ON567195**	**ON567189**	**This study**
		**CCMJ5246**	**ON567196**	**ON567190**	
		**CCMJ5247**	**ON567197**	**ON567191**	
		**CCMJ5248**	**ON567198**	**ON567192**	
		**CCMJ5249**	**ON567199**	**ON567193**	
		**CCMJ5250**	**ON567200**	**ON567194**	
*T. ghanense*	*Agaricus bisporus*	NBRC30902	AB807638	—	[76]
*T. hamatum*	*Agaricus bisporus*	Tham20-3	—	—	[81]
	*Lentinula edodes*	—	—	—	[82]
	—	DAOM167057 (T)	EU279965	AF545548	[57]
	—	Hypo647 = WU31629	KJ665513	KJ665274	[20]
	—	Hypo648 = CBS132565	KJ665514	KJ665275	[20]
*T. harzianum*	*Pleurotus ostreatus*	KACC40558	—	—	[66]
	*Cyclocybe aegerita*	JB1	—	—	[73]
	*Lentinula edodes*	T50	—	—	[83]
	*Pleurotus eryngii*	KACC40784	—	—	[69]
	*Pleurotus ostreatus*				
	*Agaricus bisporus*	—	—	—	[45]
	*Pleurotus ostreatus*	—	—	—	[84]
	Polypores/Corticiaceous	—	—	—	[18]
	*Pleurotus tuoliensis*	—	—	—	[85]
	*Tremella fuciformis*				
	*Flammulina filiformis*				
	—	CBS226.95	AF348101	AF545549	[48]
	—	Thaum12	MT081433	MT118248	[86]
	—	CBS227.95	AF348100	—	[45]
	—	GJS05-107	FJ463329	FJ442708	In GenBank
	—	GJS04-71	FJ463396	FJ442779	In GenBank
*T. hengshanicum*	*Ganoderma sichuanense*	1009	—	—	[87]
	—	HMAS248852 (T)	KY688054	KY687991	[47]
	—	HMAS248853	KY688055	KY687992	
*T. inhamatum*	*Agaricus bisporus*	CBS273.78 (T)	AF348099	FJ442725	[81]
	*Pleurotus tuoliensis*	—	—	—	[85]
*T. koningii*	*Pleurotus eryngii*	—	—	—	[69]
	*Agaricus bisporus*	—	—	—	[88]
	*Lentinula edodes*	—	—	—	[85]
	*Pleurotus ostreatus*				
	*Pleurotus tuoliensis*				
	*Flammulina filiformis*				
	*Volvariella volvacea*				
	*Hypsizygus marmoreus*				
	*Ganoderma sichuanense*	TFl040917	—	—	[75]
	*Tremella fuciformis*	TGy040604	—	—	
	—	7723	KJ634753	KJ634720	[89]
	—	GJS90-18	DQ289007	EU248600	[23]
	—	CBS979.70	AY665703	EU248601	In GenBank
	—	S22	KC285595	KC285749	[90]
***T. koningiopsis***	*Phaiius rubrovolvata*	CXYL	MN135988	MT038997	[91]
	***Ganoderma sichuanense***	**CCMJ5253**	**ON567187**	**ON567201**	**This study**
		**CCMJ5254**	**ON567188**	**ON567202**	
*T. kunigamense*	*Lentinula edodes*	TAMA193	AB807645	AB807657	[76]
*T. leguminosarum*	dark corticiaceous fungus	S391	KJ665548	KJ665287	[20]
		CBS130014	KJ665551	KJ665288	
		S503	KJ665552	KJ665289	
*T. lieckfeldtiae*	*Moniliophthora roreri*	GJS00-14 = CBS123049 (T)	EU856326	EU883562	[92]
*T. longibrachiatum*	*Pleurotus ostreatus*	TUFC61535 = CBS816.68(T)	EU401591	DQ087242	[40]
	*Agrocybe aegerita*	JB4	—	—	[73]
	*Lentinula edodes*	T57	—	—	[83]
	*Ganoderma sichuanense*	TFl040921	—	—	[75]
	*Pleurotus eryngii*	—	—	—	[93]
	*Agaricus bisporus*	—	—	—	[81]
	*Pleurotus tuoliensis*	—	—	—	[85]
	*Hypsizygus marmoreus*				
	*Volvariella volvacea*				
*T. mienum*	*Lentinula edodes*	TUFC61517	JQ621975	JQ621965	[94]
*T. orientale*	*Ganoderma applanatum*	LESF516	KT279041	KT278976	[46]
	*Ganoderma applanatum*	LESF540	KT279042	KT278977	
	*Ganoderma applanatum*	LESF544	KT279043	KT278978	
	*Ganoderma applanatum*	TRS707	KP008888	KP009202	
*T. oblongisporum*	*Lentinula edodes*	T37	—	—	[83]
	—	DAOM167085	AF534623	AF545551	[48]
*T. parareesei*	*Pleurotus eryngii*	TAMA0153	AB807640	AB807652	[76]
*T. parestonica*	*Hymenochaete tabacina*	CBS120636 (T)	FJ860667	FJ860565	[18]
*T. pleuroticola*	*Pleurotus ostreatus*	CBS124383 (T)	HM142381	HM142371	[66]
		CPK2885	EU918161	EU918141	
	*Pleurotus eryngii*	CAF-TP3	—	—	[69]
	*Lentinula edodes*	T22	—	—	[83]
	*Cyclocybe aegerita*	JB7	—	—	[73]
	—	T1295	EU279973	—	[57]
*T. pleuroti*	*Pleurotus ostreatus*	KACC44537	—	—	[69]
	*Pleurotus eryngii* var. *ferulae*	—	—	—	[95]
	—	CBS124387 (T)	HM142382	HM142372	[50]
*T. polypori*	*Lentinula edodes*	HMAS248861	KY688059	KY688000	[47]
	*Polyporus* sp.	HMAS248855 (T)	KY688058	KY687994	
*T. polysporum*	*Lentinula edodes*	—	—	—	[96]
	—	8232	KJ634779	KJ634746	[89]
	—	8147	KJ634771	KJ634738	
*T. priscilae*	*Crepidotus* sp.	S168 = CBS131487 (T)	KJ665691	KJ665333	[20]
	*Stereum* sp.	S129	KJ665689	KJ665332	
	—	HMAS245002	KT343760	KT343764	In GenBank
*T. protopulvinatum*	*Fomitopsis pinicola*	CPK2434	FJ860677	FJ860574	[18]
*T. pulvinatum*	*Fomitopsis pinicola*	CBS121279	FJ860683	FJ860577	[18]
*T. pseudokoningii*	*Lentinula edodes*	DUCC4021	KX431217	—	[77]
	*Cyclocybe aegerita*	TGc050619	—	—	[75]
	*Ganoderma sichuanense*	TFl040926	—	—	
	*Pleurotus eryngii*	—	—	—	[97]
	*Flammulina filiformis*	—	—	—	[98]
	*Pleurotus tuoliensis*	—	—	—	[85]
	*Volvariella volvacea*				
	*Hypsizygus marmoreus*				
	—	DAOM167678	AY865641	KJ842214	[99]
	—	GJS99-149	JN175589	JN175536	[17]
	—	GJSNS19	JN175588	JN175535	
*T. pseudolacteum*	*Lentinula edodes*	TUFC61496	JX238494	JX238479	[100]
		TUFC61502	JX238480	JX238471	
*T. samuelsii*	*Hymenochaete* sp.	S5 = CBS130537	JN715651	JN715599	[101]
		S42	JN715652	JN715598	
*T. songyi*	*Tricholoma matsutake*	TC556	KX266244	KX266250	[102]
		TC480	KX266243	KX266249	
*T. stilbohypoxyli*	*Stilbohypoxylon moelleri*	Hypo256 = CPK1977	FJ860702	FJ860592	[23]
*T. stromaticum*	*Agaricus bisporus*	GJS97-181	AY937447	HQ342227	[59]
	—	GJS07-88	HQ342195	HQ342258	[103]
	—	GJS03-47	HQ342201	HQ342264	
	—	GJS00-107	HQ342202	HQ342265	
*T. sulphureum*	*Laetiporus sulphureus*	CBS119929	FJ860710	FJ179620	[18]
		CPK1593	FJ860709	FJ860599	
	*Thelephora* sp.	GJS95-135 = CBS114237	AY392006	AY391958	[53]
*T. tsugarense*	*Lentinula edodes*	TAMA203 (T)	AB807647	AB807659	[76]
*T. viride*	*Lentinula edodes*	T13	—	—	[83]
	*Pleurotus ostreatus*	—	—	—	[82]
	*Tremella fuciformis*	TGc040905	—	—	[75]
	*Ganoderma sichuanense*	TFl080706	—	—	[75]
	*Flammulina filiformis*	TFj10010	—	—	[75]
	*Cyclocybe aegerita*	TGc040905	—	—	[75]
	*Phallus indusiatus*	TFl080706	—	—	[75]
	*Tremella fuciformis*	TGc040905	—	—	[75]
	*Agaricus bisporus*	—	—	—	[88]
	*Pleurotus eryngii*	—	—		[69]
	—	TRS575	KP008931	KP009081	In GenBank
	—	LESF115	KT278989	KT278921	[46]
*T. virens*	*Agaricus bisporus*	—	—	—	[88]
	*Pleurotus eryngii*	—	—	—	
	—	DIS162	FJ463367	FJ442696	In GenBank
	—	DIS328A	FJ463363	FJ442738	In GenBank
*T.* cf. *virens*	*Pleurotus eryngii*	KACC40783	—	—	[69]
	*Pleurotus ostreatus*	TUCIM2558	KX655776	—	[104]
*T. viridarium*	*Steccherinum ochraceum*	GJS89-142	AY376049	EU241495	[51]
	*Nemania* sp.	GJS98-182	DQ307511	EU252011	[23]
*Protocrea farinosa*	—	CBS121551	EU703889	EU703935	[105]
*Protocrea pallida*	—	CBS121552	EU703897	EU703944	

The type sequences are marked with (T), the new sequences are shown in bold.
